# Feasibility Study of Intratumoral NRF2 Expression as a Predictive Biomarker for the Effectiveness of Immunotherapy in Patients with Non-Small Cell Lung Cancer Treated with PD-1 Inhibitor

**DOI:** 10.3390/cancers18142202

**Published:** 2026-07-08

**Authors:** Yasuto Jin, Yukihisa Inoue, Hiroyuki Shimada, Tetsu Hara, Shohei Yamashita, Mio Yamamoto, Osamu Matsubara

**Affiliations:** 1 Department of Respiratory Medicine, Hiratsuka Kyosai Hospital, 9-11 Oiwake, Hiratsuka 254-8502, Kanagawa, Japan; inoue-yk@kkr.hiratsuka.kanagawa.jp (Y.I.); shimada-h@kkr.hiratsuka.kanagawa.jp (H.S.); hara-t@kkr.hiratsuka.kanagawa.jp (T.H.); yamashita-sh@kkr.hiratsuka.kanagawa.jp (S.Y.); yamamoto-mi@kkr.hiratsuka.kanagawa.jp (M.Y.); 2 Department of Diagnostic Pathology, Hiratsuka Kyosai Hospital, 9-11 Oiwake, Hiratsuka 254-8502, Kanagawa, Japan; matsubara-o@kkr.hiratsuka.kanagawa.jp

**Keywords:** non-small cell lung cancer, immune check point inhibitor, cancer immunotherapy, metabolic reprogramming, NRF2, predictive factor, reactive oxygen species

## Abstract

**Background**: Overexpression of programmed death-ligand 1 (PD-L1) and programmed cell death protein 1 (PD-1) induces immune evasion by cancer cells. Nivolumab and pembrolizumab (anti-PD-1 antibodies) are used to treat advanced non-small cell lung cancer (NSCLC). However, objective response rates are limited (20–30%), indicating that individual tumor microenvironments may differ according to immune evasion processes. Therefore, the development of biomarkers predictive of responders to immune checkpoint inhibitors is necessary. Activation of the nuclear factor erythroid 2-related factor 2/Kelch-like ECH-associated protein 1 (NRF2/KEAP1) signaling pathway promotes lung cancer cell growth and resistance to chemotherapy, radiotherapy, targeted therapy, and PD-1/PD-L1 inhibition. The present study investigated whether NRF2 expression in NSCLC is associated with clinicopathological factors, the expression levels of intratumoral PD-L1 and CD8, and the efficacy of anti-PD-1 monotherapy. **Methods**: NRF2, PD-L1, and CD8 expression on tumor cells and tumor-infiltrating lymphocytes were examined by immunohistochemistry in 54 patients with advanced adenocarcinoma (*N* = 40) and squamous cell carcinoma (*N* = 14) treated with nivolumab or pembrolizumab. Histological subtypes, tumor stages, and other clinicopathological features were compared with their expression levels. **Results**: Weak NRF2 staining was significantly correlated with high levels of PD-L1 and CD8+ tumor-infiltrating lymphocytes, and a favorable response to treatment with nivolumab or pembrolizumab in NSCLC. Progression-free survival of patients treated with anti-PD-1 therapy differed according to the different NRF2 levels. **Conclusions**: NRF2 overexpression in NSCLC is associated with resistance to PD-1 blockade monotherapy.

## Simple Summary

Cancer immunotherapy with programmed cell death protein 1 (PD-1) inhibitors plays an important role in the treatment of non-small cell lung cancer; however, its efficacy remains limited. Metabolic reprogramming in cancer cells generates reactive oxygen species and an immunosuppressive tumor microenvironment. Nuclear factor erythroid 2-related factor 2 (NRF2) is a major factor in the control of cytoprotective genes linked to redox homeostasis. In normal cells, expression of NRF2 is transient. In non-small cell lung cancer, overexpression of NRF2 is common and associated with treatment resistance and cancer cell proliferation. A simple and conventional technique to detect the NRF2 expression may be helpful for the combined use of biomarkers with immunotherapy.

## 1. Introduction

Cancer immunotherapy plays an important role in the treatment of numerous types of cancer, including lung cancer [1]. Inhibitors of the programmed cell death protein 1/programmed death-ligand 1 (PD-1/PD-L1) pathway may reverse the function of exhausted T cells and enhance the T-cell responses of the endogenous antitumor immunity [2,3,4]. Effector memory T cells in the tumor microenvironment (TME) increase in patients treated with immune checkpoint inhibitors (ICIs) who respond to treatment [5]. However, the clinical efficacy for prognosing the precise benefit of ICIs is limited, with objective response rates of approximately 20% in all patients with advanced non-small cell lung cancer (NSCLC) treated with PD-1 blockade monotherapy [1,6]. It has been clinically documented that the expression status of PD-L1 on tumor cells and tumor mutational burden are predictive of favorable response [7,8,9,10]. In previous studies, the response rates in patients with at least 50% PD-L1 positivity on the tumor were 45% [11,12]. The TME immune type based on PD-L1 status and tumor-infiltrating lymphocytes (TILs) in lung adenocarcinoma and squamous cell carcinoma is correlated to patient prognosis [13,14,15]. The balance of PD-1 expression between CD8+ effector cells and regulatory T (Treg) cells in the TME is a significant predictor of immunotherapy by PD-1 blockade [16,17,18,19]. Metabolite-directed changes support anabolic reactions and high redox homeostasis of cancer cells and alter the immune profile and cell behavior in the TME. This process results in poor response to immunotherapy in terms of reducing the activity of infiltrating CD8+ T cells and the number of tissue-resident memory CD8+ T cells, as well as enhancing Treg cell function [20,21,22,23,24,25].

Excess oxidative stress beyond physiological levels caused by reactive oxygen species (ROS), the most common reactive species, damages macromolecules [26]. ROS are generated in mitochondria (the main source of oxidative stress) as products of oxidative metabolism. Cancer cells have multiple defense mechanisms against oxidative stress, characterized by the development of therapeutic resistance. Endogenous antioxidants, such as glutathione (GSH), are used to neutralize the ROS in tumor cells and the TME [27,28]. Nuclear factor erythroid 2-related factor 2 (NRF2) pathways are major systems for maintaining cellular redox homeostasis and cell survival by the induction of phase II detoxifying enzymes and antioxidant enzymes.

Large-scale clinical database analysis showed that co-mutation of KRAS oncogenic genes, Kelch-like ECH-associated protein 1 (KEAP1), SWI/SNF related BAF chromatin remodeling complex subunit ATPase 4 (SMARCA4), serine/threonine kinase 11 (STK11) genes, and clonal KEAP1 gene mutations with loss of heterozygosity enhance tumor biology, cause metabolic reprogramming, increase the aggressiveness, proliferation, and therapy resistance of NSCLC, and shorten the progression-free survival (PFS) and overall survival (OS) of patients treated with ICIs, independent of PD-L1 levels [29,30,31,32,33,34,35,36].

KEAP1 is an adaptor molecule of NRF2 and cullin 3 (CUL3), a subunit of the E3-ubiquitn ligase, a thiol-based sensor of ROS, and a regulator of NRF2. In oxidative stress response, newly synthesized NRF2 accumulates and translocates to the nucleus [37,38]. Target genes of NRF2 relate to adaptation to redox homeostasis, drug metabolism, metabolic reprogramming, cell survival, and proliferation [39]. Under normal conditions, activation of NRF2 is transient and typically maintained at low levels; this mechanism is beneficial to normal cells. Under pathological conditions, the NRF2-KEAP1 system plays an important role in the proliferation of tumor cells in various types of human cancers [40,41].

In lung cancer cells, loss of KEAP1 function induces constitutive NRF2 upregulation and the activation of its target genes. Regarding KEAP1, its mutations are scattered throughout the genome length [42]. Its mutation patterns are tumor-suppressive in manner, and some KEAP1 gene variants exhibit dominant-negative effect. And clonal KEAP1 gene mutations with loss of heterozygosity were reported [43]. Furthermore, a higher level of *CpG* island methylation within the promoter or exon1 region of *KEAP1* causes epigenetic mechanisms [44,45,46]. The accumulation of NRF2 at high levels induces metabolic reprogramming which changes the cellular redox status and purine nucleotide synthesis is associated with poor prognosis in various types of cancer, including lung cancer [47,48,49,50,51,52,53,54,55,56,57,58]. NRF2 is thought to be a tumor suppressor as part of cyto-protection in normal cells as well as in premalignant and early malignant cells [59]. The enhancement of NRF2 activity caused by genetic mutations can protect tumor cells from the cytotoxic effects of ROS induced by tumor cells, chemotherapy, and radiotherapy, thus facilitating tumor progression [60]. The frequency of KEAP1 and NRF2 gene mutations in NSCLC are 11–20% and 3.5–30% in European ancestry, and 0.5% and 13.6% in East Asian ancestry, respectively [61]. In Japanese large-scale cohort study, it was reported that KEAP1 and NRF2 variants were 2.5% and 19.6%, respectively, and that each type exhibits different biological characteristics according to the somatic mutations [62]. KEAP1 gene mutations are predominant in adenocarcinoma, whereas NRF2 gene mutations are slightly more frequent in squamous cell carcinoma, and both mutations are almost exclusive [62]. The development of therapy resistance by activation of the KEAP1/NRF2 pathway through other genomic or epigenetic changes and metabolic mediators has been reported [60,63,64,65,66,67,68].

Various molecular mechanisms of NRF2 activation, such as alteration of KEAP1, NRF2, or CUL3 genes, gene copy number alterations, accumulation of phosphorylated p62, p21 interaction with the Neh2 domain of NRF2, epigenetic mechanisms of KEAP1 gene regulation, NRF2 phosphorylation at multiple sites under the signaling of mitogen-activated protein kinase (MAPK) and PI3K/AKT pathways, and glycogen synthase kinase 3 (GSK-3)-phosphorylation-dependent degradation in a KEAP1-independent manner, have been reported [45,46,68,69,70,71,72]. The metabolic phenotypes of KEAP1/NRF2 were categorized by their gene expression and other mutations (e.g., STK11 and SMARCA4 genes) [24]. Recently, the metabolic phenotype of KEAP1/NRF2 gene-mutated NSCLC was reported in NSCLC without KEAP1/NRF2 gene mutation, which was estimated to represent 10% of KEAP1/NRF2 activation phenotype [73].

The relationship between KEAP1/NRF2 alterations and response to immune therapy has been investigated in numerous studies. However, the relationship between overexpression of NRF2 protein (including wild type) and response to immunotherapy remains unknown [74,75,76,77,78,79].

In Japan, comprehensive genomic profiling (CGP) tests have been available under the national health care system only for patients with solid cancers who have completed standard treatment [80]. Wang et al. reported that their meta-analysis indicated the significance of the prognostic value of NRF2 in solid tumors, and higher expression of NRF2 detected by immunohistochemistry related to worse disease-free survival and overall survival [81]. Thus, the present study retrospectively evaluated the expression of NRF2 protein, PD-L1 levels, and CD8+ TIL density by immunohistochemistry (IHC) in patients with advanced NSCLC treated with an anti-PD-1 monoclonal antibody (i.e., nivolumab or pembrolizumab). The objective was to determine whether the expression of these molecules was associated with favorable response to the ICIs, PFS, and OS.

## 2. Materials and Methods

### 2.1. Patient Data

The study included 54 patients with advanced NSCLC who received ICI (nivolumab or pembrolizumab) monotherapy at any line of treatment at the Hiratsuka Kyosai Hospital (Hiratsuka, Japan) between 2016 and 2018. Clinicopathological features of the patients are summarized in Table 1. Most patients were current or former smokers. Pathological classification of the tumors followed the World Health Organization criteria [82]. Of the 54 patients, 40 and 14 had adenocarcinoma and squamous cell carcinoma, respectively. Clinical TNM staging was performed according to the second edition of The International Association for the Study of Lung Cancer Staging Manual in Thoracic Oncology [82]. The clinical stage in this group was stage IIIB in nine patients and stage IV in 45 patients. Few patients had epidermal growth factor receptor-mutant (EGFR-mutant) or anaplastic lymphoma kinase-translocated (ALK-translocated) tumors. The research protocol was conducted in accordance with the tenets stipulated in the Declaration of Helsinki and approved by the Institutional Ethics Review Board (approval number: 4-24; date of approval: 7 March 2023).

### 2.2. IHC Analysis

IHC on the Ventana BenchMark platform was performed using the setting recommended by the manufacturer. Formalin-fixed, paraffin-embedded tissue blocks were cut into slices (thickness: 4 μm). Anti-NRF2 rabbit polyclonal antibody (Proteintech Group, Inc., Rosemont, IL, USA), VENTANA PD-L1 (SP263) rabbit monoclonal antibody (Ventana Medical Systems, Inc., Tucson, AZ, USA), and CONFIRAM anti-CD8 (SP57) rabbit monoclonal antibody (Ventana Medical Systems, Inc.) were used as primary antibodies. Hematoxylin was used as counterstain. Negative control sections were prepared as described above, omitting the incubation with the primary antibody. Positive controls consisted of non-neoplastic epithelial cells of the proximal or distal airway.

**Figure 1:**
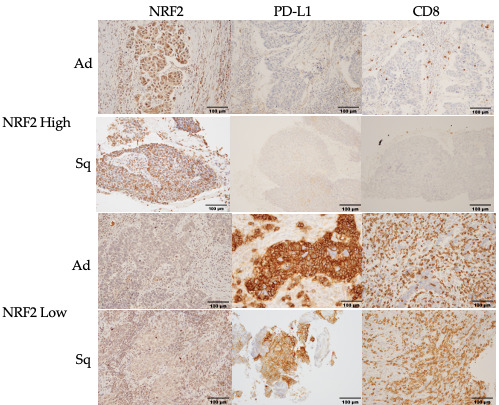
Immunohistochemical staining of NRF2, PD-L1, and CD8 in lung tumors. Immunoreactivity for NRF2 was observed in the nucleus or cytoplasm. Tumor cells with reduced expression of NRF2 showed strong expression of PD-L1 at the tumor cell membrane and abundant CD8+ lymphocyte infiltrates in the tumor tissue. Ad, adenocarcinoma; NRF2, nuclear factor erythroid 2-related factor 2; PD-L1, programmed death-ligand 1; Sq, squamous cell carcinoma. Scale bar: 100 μm.

**Figure 2:**
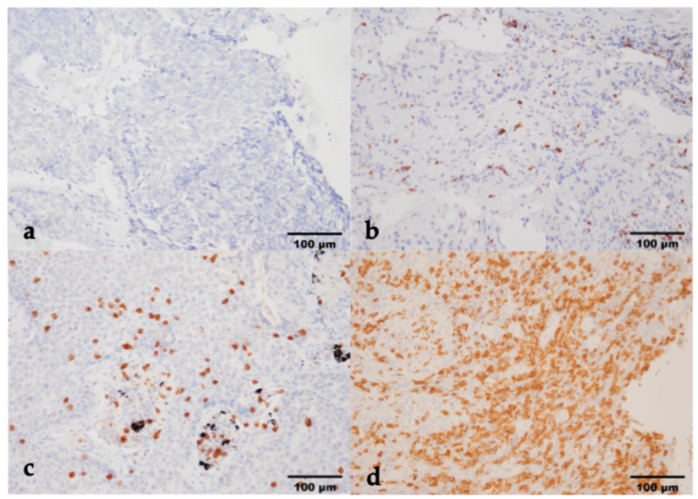
The presence of CD8+ cells in the cancer cell nest was assessed as follows: (**a**), (**b**), (**c**), and (**d**) were scored as 0, I, II, and III, indicating 0, 1−5, 6−24, and >25, respectively.

NRF2 staining was scored as previously described [83]. Positive immunostaining of NRF2 was defined as similar or stronger intensity of NRF2 staining in the nucleus or cytoplasm of tumor cells compared with that detected in normal bronchial epithelial cells. The degree of NRF2 expression is presented either as low level (0–49% positive tumor cells) or high level (50–100% positive tumor cells). PD-L1 expression in tumor cells was evaluated by the degree of membrane staining as follows: the whole section was negative, between 1 and 49%, and ≥50% [84,85,86]. The degrees of PD-L1+ tumor cells were classified into low (0–49%) and high (50–100%) expression groups. To determine the presence of CD8+ T cells within cancer cell nests and cancer-associated stroma, we counted the number of immunoreactive CD8+ cells within a microscopic field of ×200 (0.01 mm^2^). The degrees of PD-L1+ tumor cells were classified into low (0–49%) and high (50–100%) expression groups (Figure 1). Three areas with the greatest abundance of CD8+ cells were selected, and average numbers of 0, 1−5, 6−24, and >25 were scored as 0, I, II, and III, respectively (Figure 2). The CD8+ cells were classified into high (II, III) or low (0, I) expression groups (Table 2).

### 2.3. Statistical Analysis

Fisher’s exact test was used for statistical analyses between the degree of IHC staining and clinicopathological categorical factors. Non-normally distributed variables were compared using the two-sided Mann–Whitney *U* test. Two-sided *p*-values <0.05 indicated statistically significant differences. Survival curves were produced by univariate analysis according to the Kaplan–Meier method, and *p*-values were calculated by log-rank testing of the univariate comparison of PFS and OS between the two groups. Multivariate Cox regression analysis was performed to adjust for potential confounding factors, including age and ECOG Performance Status score, calculating hazard ratios with 95% confidence intervals. All analyses were performed using GraphPad Prism version 11 (GraphPad, Inc., San Diego, CA, USA). PFS and OS were measured from the time of immunotherapy initiation to investigator-assessed radiographic progression or death, respectively.

## 3. Results

### 3.1. Association of NRF2, PD-L1, and CD8 Expression

Positive immunostaining of NRF2 was visible in the nucleus and cytoplasm in lung tumor sections (Figure 1). High expression of NRF2 was detected in 29 of 54 patients (53.7%). Normal bronchi showed weak staining. PD-L1 expression at the plasma membrane was heterogeneous within the cancer cell nest. A PD-L1+ tumor was characterized by the presence of CD8+ TILs in the cancer cell nest. In contrast, a PD-L1− tumor showed CD8+ TILs in the cancer-associated stroma rather than in the cancer cell nest (Figure 2). Lower PD-L1 levels and numbers of CD8+ TILs were observed in the area with high NRF2 expression in the tumor (Figure 2).

### 3.2. Response to Treatment with ICI in Patients with Gene Alteration

Immunotherapy is usually less effective for actionable driver gene mutations and is reserved for later-line treatment. Patients received prior targeted therapies including second- and third-line treatment. The efficacy of mono-immunotherapy for patients with a driver oncogene and the expression levels of NRF2 and PD-L1 were listed in Table 2. There was no correlation between PFS and OS.

**Figure 3:**
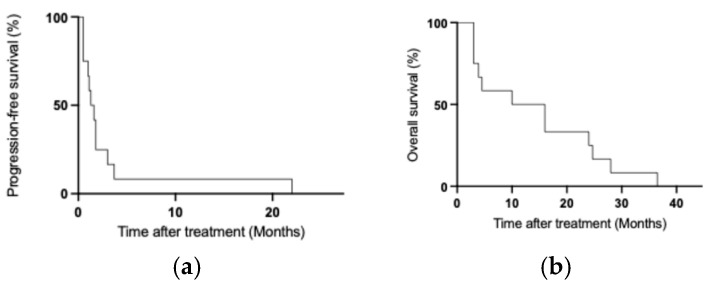
Kaplan–Meier curves of (**a**) progression-free survival and (**b**) overall survival after treatment in patients with a driver gene mutation.

Kaplan–Meier curves of PFS and OS are shown in Figure 3. Cox proportional-hazard model was used to estimate the hazard ratio (for NRF2 High as compared to Low).

**Figure 4:**
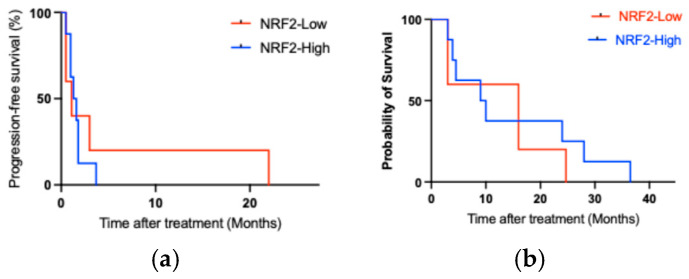
Kaplan–Meier curves of (**a**) progression-free survival and (**b**) overall survival after treatment in subgroups of patients with driver-gene mutations according to NRF2 levels with the percentage of staining.

Then we evaluated whether the degree of tumor cells with high NRF2 staining correlated with PFS and OS. There was no significant difference between the two groups of patients with driver mutations in the survival curves (PFS: median 1.10 versus 1.45 months, respectively, hazard ratio: 1.255, 95% confidence interval: 0.4198–3.752, *p* = 0.6486; OS: median 16.0 versus 9.5 months, respectively, hazard ratio: 0.7567, 95% confidence interval: 0.2362–2.424, *p* = 0.5871). (Figure 4a,b) The three patients with ECOG PS 2 may have tended to have shorter OS (less than 12 months), but there was no significant difference between PS 0/1 and 2. Multivariate Cox proportional hazards regression analysis was performed for both PFS and OS to evaluate the independent prognostic value of NRF2 expression after adjusting for gene alterations and ECOG PS. In the multivariate Cox regression analysis, low NRF2 expression was identified as independently associated with prolonged OS (hazard ratio: 0.1395; 95% confidence interval: 0.007476–0.7568) after adjusting for gene alterations and ECOG PS (Table 3).

### 3.3. Response to Treatment with ICI Based on the Levels of NRF2 Expression

There was no significant association of NRF2 with smoking status, tumor histology, clinical staging, and lines of treatment in these patients; however, increased NRF2 expression was observed in older patients (Table 4). There were more male smokers than female smokers (Table 1). Inverse correlations between the levels of NRF2, PD-L1 expression, and the number of the CD8+ TILs were determined (Table 4).

### 3.4. PFS and OS

**Figure 5:**
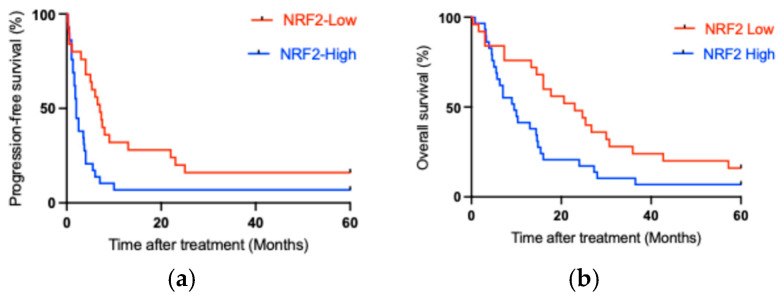
Kaplan–Meier curves of (**a**) progression-free survival and (**b**) overall survival after treatment in subgroups of patients according to NRF2 levels. Progression-free survival and overall survival were significantly longer in patients with low expression of NRF2 than in those with high expression. NRF2, nuclear factor erythroid 2-related factor 2.

After 60 months of follow-up, four (16%) and two (7%) patients with low and high NRF2 expression, respectively, were alive. The patients with low NRF2 expression levels had a significantly better PFS and OS than those with high NRF2 expression levels (PFS: median 7.3 versus 2.0 months, respectively, hazard ratio: 0.45, 95% confidence interval: 0.25–0.81, *p* = 0.0027; OS: median 23 versus 9.5 months, respectively, hazard ratio: 0.53, 95% confidence interval: 0.30–0.94, *p* = 0.022) (Figure 5a,b).

### 3.5. Correlation Between NRF2 Staining and OS According to the NRF2 Expression Levels with the Percentage of Staining

**Figure 6:**
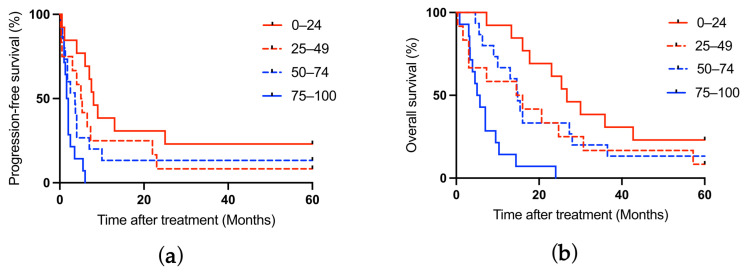
Kaplan–Meier curves of (**a**) progression-free survival and (**b**) overall survival after treatment in subgroups of patients according to NRF2 levels with the percentage of staining. Progression-free survival and overall survival were significantly shorter in patients with higher expression (75–100%) of NRF2 than in those with other categories. NRF2, nuclear factor erythroid 2-related factor 2.

Subsequently, we evaluated whether the percentage of tumor cells staining positive for NRF2 correlated with PFS and OS. The staining was categorized as follows: 0–24% (*n* = 13), 25–49% (*n* = 12), 50–74% (*n* = 15), and 75–100% (*n* = 14). A total of 14 patients had 75–100% tumor cells that stained positive for NRF2. The patients with higher NRF2 expression levels (75–100%) had a significantly shorter PFS (*p* = 0.0014) and OS (*p* < 0.0001) than those with lower NRF2 levels (Figure 6a,b). Multivariate Cox proportional hazards regression analysis was performed for both PFS and OS to evaluate the independent prognostic value of NRF2 expression after adjusting for age and ECOG PS. In the multivariate Cox regression analysis, both age (HR 0.9533, 95% CI: 0.9166–0.9941) and low NRF2 expression (HR 0.1378, 95% CI: 0.005035–0.916) were independently associated with prolonged OS after adjusting for PS (Table 5). The results suggest that high expression of NRF2 affects poor OS.

## 4. Discussion

The results of this feasibility study demonstrated that overexpression of NRF2 is related to lower levels of PD-L1 in tumor cells and CD8+ TILs, as well as revealed the association of NRF2 expression with significantly worse OS. The patients with a higher percentage (75–100%) of tumor staining positive for NRF2 had a significantly lower survival rate than those of patients with 0–74%. The patients with 25–49% and 50–74% of tumor cell staining positive for NRF2 indicated a similar tendency. Durable responses and long-term survival were observed in patients with <75% positive staining who were previously treated for advanced NSCLC. Therefore, levels of NRF2 may be useful as a prognostic biomarker or a predictive biomarker for response to treatment with ICIs, as well as indicate resistance to immunotherapy.

The potential limitations are the high heterogeneities, because the number of samples was small, which is not sufficient to detect remarkable differences between each characteristic, and the lack of stratified analysis for different pathological tumor types. Other limitations of this study should be acknowledged. First, our findings lack large-scale validation. Second, the inter-rater reliability of NRF2 scoring was not evaluated using Cohen’s kappa statistic, which may introduce potential subjectivity. Furthermore, this study did not test gene expression profiles or perform multiplex immunohistochemistry (IHC) and spatial analyses, which are necessary to comprehensively understand the underlying molecular mechanisms.

In this study, all patients with oncogene-addicted NSCLC received effective targeted therapies, including chemotherapy in some cases. Some patients showed survival for more than 2 years. However, ICI therapy for patients with oncogene-addicted NSCLC remains controversial [87]. Chen et al. reported that the pooled median PFS and median OS following immunotherapy were 2.33 and 12.43 months, respectively [88]. Our results indicated a similar tendency (Figure 3a,b).

Jeong et al. reported that in 51 patients with stage IV harboring *KEAP1*/*NFE2L2*/*CUL3* mutations who received first-line platinum doublet chemotherapy, time to treatment failure and overall survival were 2.8 months and 11.2 months, respectively. These were significantly shorter than those of characteristic-matched 52 patients with 52 *KEAP1*/*NFE2L2*/*CUL3* wild-type tumors [89]. Duan reported that in a large-scale clinical trial (*N* = 360) for advanced squamous cell carcinoma, a prolonged PFS benefit in the tislelizumab plus chemotherapy arm versus the chemotherapy arm was observed in patients with was noted in patients with low NRF2 levels, whereas reduced benefit was noted in those with high NRF2 levels. These results suggest that patients harboring activating mutations in the NRF2 pathway tend to be resistant to combination therapy [79]. Further characterization of immune cell populations revealed lower expression of T-cell gene signatures.

Gene expression signatures in tumor tissue, which are linked to the tumor immune microenvironment, have been explored as potential alternative biomarkers. NRF2 signature scores and PD-L1 expression were correlated in patients with low gene expression signatures, and NRF2 mediated constitutive PD-L1 expression was observed in the non-inflamed microenvironment. Combination biomarkers were efficient in identifying the patients with PD-L1 negative and NRF2-high tumor who did not benefit from tislelizumab plus chemotherapy, whereas those with PD-L1-negative and NRF2-low tumors had a modest PFS benefit.

The predictive value of PD-L1 as a biomarker for response to immunotherapy in patients with lung cancer has been previously discussed [84,90]. Assay variability and tumor heterogeneity are the most common limiting factors for biomarker generalizability in clinical settings, because biomarker detection is based on a small biopsy specimen of cancer tissue obtained at a single time point. The detection of PD-L1 protein by IHC is used as a companion diagnostic test. Moreover, IHC using antibodies, namely the 28-8 (Dako, Glostrup, Denmark), 22C3 (Dako), and SP263 (Ventana Medical Systems, Inc.) assays, for PD-L1 expression on tumor cells have demonstrated interchangeability in predicting response to treatment with nivolumab and pembrolizumab in patients with NSCLC [5,85]. The small biopsy specimens may not represent the whole tumor because the TME is heterogeneous and immune cell infiltration into tumors varies between cases. Kawasaki et al. reported the significance of NRF2 IHC scoring as a simple and widely used method in clinical practice for predicting prognosis and treatment response to chemoradiotherapy in patients with esophageal squamous cell carcinoma [91]. Nakayama et al. reported that resistance to cisplatin is induced by NRF2-axis activation in cell lines; moreover, in head and neck squamous cell carcinoma, immunohistochemical analysis of clinical samples revealed that high expression was associated with poor prognosis after cisplatin-based chemoradiotherapy [92]. Islam et al. reported that positive nuclear immunostaining for NRF2 in tumor cells with KEAP1 mutations (i.e., SSC9 and KEAP1-mutated patient tumor cells) demonstrated increased nuclear localization of NRF2 compared with normal and KEAP1 wild-type cells [93]. In this feasibility study, patients with higher NRF2 expression levels had a significantly shorter PFS and OS than those with lower NRF2 levels. Therefore, these results may be helpful in predicting response to treatment with PD-1 axis inhibitors. In clinical practice, detection of the protein through IHC is a simple and effective biomarker for the treatment of lung cancer. In the laboratory, gene sets representing NRF2 activation have been investigated for the prediction of drug resistance. The International Association for the Study of Lung Cancer Pathology Committee provided an overview of immunotherapy biomarkers, including a multimodal approach such as multiplex immunofluorescence [94]. Highly multiplexed, spatially resolved technology enables the simultaneous analysis of numerous markers.

Several new therapeutic approaches targeting the redox system, such as direct inhibition of NRF2, indirect approaches targeting the KEAP1/NRF2 pathway, inhibitors of mTOR and PI3K pathways, glutamine-pathway inhibitors, the combination of a glutaminase inhibitor with ICIs, and inhibitors of ROS scavengers, have been proposed [54,77,95,96,97,98,99,100,101,102,103]. Nrf2 activation in immune cells in the tumor microenvironment suppressed Nrf2-activated cancer progression [104]. These reports indicate the importance of estimating the expression of NRF2 levels in clinical practice.

## 5. Limitation of This Study

This observational study was conducted at a single institution and included a small number of patients. Small sample sizes increase the risk of random error and overestimation. Further independent and prospective studies are needed. Due to the limited sample size and lack of information on gene profiles of the KEAP1/NRF2 pathway, it is possible that biomarkers were overlooked. Nevertheless, the relationship between NRF2 overexpression and the efficacy of treatment is worthy of further exploration.

## 6. Conclusions

The present evidence suggests that overexpression of NRF2 in NSCLC is linked to resistance to PD-1 blockade in patients with NSCLC.

## Figures and Tables

**Table 1:** Basic patient characteristics (*N* = 54).

Characteristic	Number/Mean ± Standard Deviation
**Sex**	
Male	40
Female	14
**Age, years**	68.7 ± 8.648
**Smoking status**	
Never	9
Current/Former	45
**ECOG Performance Status score**	
0/1	45
2	9
**Histology**	
Adenocarcinoma	40
Squamous cell carcinoma	14
**Tumor stage at diagnosis**	
III	9
IV	45
**Gene alterations**	
EGFR mutation	12
ALK rearrangement	1
**ICI monotherapy treatment**	
First line	12
Second line	9
Third line or higher	33

Abbreviations: ALK, anaplastic lymphoma kinase; ECOG, Eastern Cooperative Oncology Group; EGFR, epidermal growth factor receptor; ICI, immune checkpoint inhibitor.

**Table 2:** Expression levels of NRF2 and ECOG PS, PFS, and OS in NSCLC patients with driver-gene mutation (*N* = 13).

Gene Alteration	NRF2	ECOG PS	PFS (Month)	OS (Month)
**EGFR mutation**				
Ex19 del	High	2	1	3
Ex 19 del	Low	1	1.1	16
Ex 21 L858R, Ex 20 T790M	High	0	1.8	36.5
Ex 21 L858R, Ex 20 T790M	High	0	1.6	4.5
Ex 21 L858R, Ex 20 T790M	High	1	1.8	28
Ex 21 L858R	High	0	1.3	24
Ex 19 G719X, Ex 20 S768I, Ex 20 T790M	Low	0	0.5	16
Ex 19 del	Low	2	3	3
Ex 21 L858R	High	0	0.5	3.9
Ex 21 L858R	Low	0	22	24.7
Ex 19 del	High	2	3.7	10
Ex 19 del	Low	1	0.5	3
**ALK rearrangement**	High	1	1	9

Abbreviation: ALK, anaplastic lymphoma kinase; ECOG, Eastern Cooperative Oncology Group; EGFR, epidermal growth factor receptor; Ex 19 del, exon 19 deletion; Ex 20, exon 20; Ex 21, exon 21; NRF2, nuclear factor erythroid 2-related factor 2; OS, overall survival; ECOG PS, ECOG Performance Status score; PFS, Progression-free survival.

**Table 3:** Multivariate analysis on prognosis of patients with non-small cell lung cancer with driver mutation.

	Progression-Free Survival	Overall Survival
	HR (95% CI)	HR (95% CI)
Gene alterations	1.031 (0.3327–3.782)	0.8921 (0.2951–3.146)
ECOG Performance Status score (0/1 vs. 2)	1.215 (0.2448–4.680)	2.781 (0.5445–11.56)
NRF2 (High vs. Low)	0.6820 (0.1510–2.249)	0.1395 (0.007476–0.7568)

Abbreviations: CI, confidence interval; ECOG, Eastern Cooperative Oncology Group; HR, hazard ratio; NRF2, nuclear factor erythroid 2-related factor 2.

**Table 4:** Clinicopathological factors associated with NRF2 expression levels in univariate analyses.

Characteristic	NRF2 Expression		Significance (*p*-Value)
	High (*N* = 29)	Low (*N* = 25)	
**Sex**			0.3443
Male	23	17	
Female	6	8	
**Age, years (mean ± SD)**	71.4 ± 9.4	66.4 ± 6.9	0.0335
**Smoking status**			0.6915
Never	3	4	
Current/Former	26	21	
**ECOG Performance Status score**			0.4802
0/1	23	22	
2	6	3	
**Histology**			0.3706
Adenocarcinoma	23	17	
Squamous cell carcinoma	6	8	
**Stage**			0.4852
III	4	6	
IV	25	19	
**Gene alteration**			>0.9999
EGFR	7	5	
ALK	1	0	
**PD-L1**			0.0278
0–49%	20	9	
50–100%	9	16	
**CD8+ cells in cancer tissue**			0.0056
High (II + III)	12	20	
Low (0 + I)	17	5	
**Overall response**			0.03831
Complete response	2	2	
Partial response	6	14	
Stable disease	8	5	
Progressive disease	13	4	
**ICI monotherapy treatment**			0.9569
First line	6	6	
Second line	5	4	
Third line or higher	18	15	

Abbreviations: ALK, anaplastic lymphoma kinase; ECOG, Eastern Cooperative Oncology Group; EGFR, epidermal growth factor receptor; NRF2, nuclear factor erythroid 2-related factor 2; ICI, immune checkpoint inhibitor; PD-L1, programmed death-ligand 1; SD, standard deviation.

**Table 5:** Multivariate analysis on prognosis of patients with non-small cell lung cancer.

	Progression-Free Survival	Overall Survival
	HR (95% CI)	HR (95% CI)
Age, year	1.019 (0.9541–1.094)	0.9533 (0.9166–0.9941)
ECOG Performance Status score (0/1 vs. 2)	1.247 (0.2806–4.029)	0.2507 (0.007823–1.876)
NRF2 (High vs. Low)	0.7044 (0.1582–2.250)	0.1378 (0.005035–0.916)

Abbreviation: ECOG, Eastern Cooperative Oncology Group; HR, hazard ratio; NRF2, nuclear factor erythroid 2-related factor 2.

## Data Availability

The original contributions presented in this study are included in the article. Further inquiries can be directed to the corresponding author.

## Abbreviations

The following abbreviations are used in this manuscript:

AKT	AKT serine/threonine kinase 1
ALK	Anaplastic lymphoma kinase
CGP	Comprehensive genomic profiling
CUL3	Cullin 3
Cys	Cysteine
ECOG	Eastern Cooperative Oncology Group
EGFR	Epidermal growth factor receptor
GSH	Glutathione
GSK3	Glycogen synthase kinase 3
ICI	Immune checkpoint inhibitor
IHC	Immunohistochemistry
KEAP1	Kelch-like ECH-associated protein 1
Maf	Musculoaponeurotic fibrosarcoma oncogene
MAPK	Mitogen-activated protein kinase
mTOR	Mechanistic target of rapamycin
NRF2	Nuclear factor erythroid 2-related factor 2
NSCLC	Non-small cell lung cancer
OS	Overall survival
PD-L1	Programmed death-ligand 1
PD-1	Programmed cell death protein 1
PFS	Progression-free survival
PI3K	Phosphoinositide 3-kinase
ROS	Reactive oxygen species
SD	Standard deviation
SMARCA4	SWI/SNF related, matrix associated, actin dependent regulator of chromatin, subfamily a, member 4
STK11	Serine/threonine kinase 11
Treg	Regulatory T
TIL	Tumor-infiltrating lymphocyte
TME	Tumor microenvironment
